# *Letter to the Editor:* Palliative Care Needs Assessment for Pakistan: Implications for Service Integration along with Policy and Practice Development

**DOI:** 10.1089/jpm.2022.0139

**Published:** 2022-06-29

**Authors:** Almas Bandeali

**Affiliations:** Development of Policies and Practices, Graduate Institute of Geneva, Geneva, Switzerland.

*Dear Editor:*

## Introduction

Palliative care (PC) takes a holistic approach in assessing and responding to patients' physical, psychological, social, spiritual, and religious needs thereby, promoting dignity, quality of life (QoL), and adjustment to progressive illnesses using the best available evidence.^1^ The World Health Assembly passed its first Resolution 67.19, titled “Strengthening of PC as a component of comprehensive care throughout the life course”^1^ for PC being human rights and government's obligation.^1^ This study aimed to gather data on Pakistan's PC services conducting a literature review (LR).

## Literature Review

World Health Organization (WHO) qualifies Pakistan as a low middle income country with 78% population requiring PC services.^2^ Sixty-three percent of Pakistan's population lives in rural areas.^3^ Seventy-three percent population depends on out-of-pocket expenditure on health-related matters.^3^ The prevalence of chronic health conditions along with socioeconomic inequalities and geopolitical instabilities not only causes limitations on the public health system in meeting the demands, but also plays a vicious role in diminishing QoL of Pakistanis.^3^ International Humanitarian Law and Human Rights obligate countries to make adequate pain medications available for all.^1,2^ LR revealed a lack of national opioid pain management policies and practices.^3^

In 2019, there was only one WHO-recognized PC program available in Karachi, Pakistan. In addition, 2020 LR revealed the passing of a bill (*PMC Act, 2020*) by the parliament, replacing Pakistan Medical and Dental Council with Pakistan Medical Commission for standardized medical and dentistry education in Pakistan, all done in accordance with the College of Physicians and Surgeons Pakistan.^4^ Furthermore, the 2021 LR confirmed nine PC-Pain Medicine fellowship programs introduced nationally to physicians interested in caring for palliative patients.^4^

## Implementations

The author recommends the following implementations: (1) To gain cost-effective sustainable momentum, incorporate PC concepts within the family medicine postgraduate residency programs. (2) To reduce health disparities related to socioeconomic inequalities and geopolitical instabilities, support public–private partnership models that enhance national health services by providing universal health coverage. (3) Introduce PC telemedicine consultations, especially for geopolitically challenged communities in Pakistan. (4) Establish PC services for patients suffering from mental health/addiction and pediatric disabilities. (5) Finally, since there is no advance care planning (ACP) nationalized policy and practice available in Pakistan, there is room for research to determine justification and attitude toward an ACP tool that may be culturally acceptable in Pakistan.

## Conclusion

PC is a human right and every state has an obligation for its citizens. Interdisciplinary research and dialogue are critical in advocating for inclusive sustainable PC policies and practices at the macro (national/governance), meso (institutional), and micro (community/clinical) levels in Pakistan. Regardless of what religion, culture, or spiritual belief we come from, there is no denying a fact that, after life is birthed, the probability of death to occur is 100%. However, the value that remains uncertain is the time of death. Just as we plan for birth, retirement, and other anticipatory events early on, we also need to raise awareness to plan for death. Pakistanis living with chronic irreversible conditions must be given care options to enhance their QoL.

## References

[1] World Health Assembly: Resolution WHA67.19 Strengthening of palliative care as a component of comprehensive care throughout the life course. 2014. https://apps.who.int/gb/ebwha/pdf_files/WHA67/A67_R19-en.pdf (Last accessed 2018).

[2] WHO Definition of Palliative Care: 2015. https://www.who.int/health-topics/palliative-care (Last accessed 2018).

[3] Masoora Maqbool Bari: Centre for Peace, Security and Developmental Studies: Dilemma of the current healthcare system of Pakistan and feasible solutions for its improvement. 2017. www.cpsd.org.pk/research-2.php (Last accessed 2018).

[4] College of Physicians and Surgeons Pakistan: Pain Medicine Accredited Programs. 2021. https://www.cpsp.edu.pk/accredited-institutes-fcps.php (Last accessed 2021).

